# Where Are Socioeconomically Deprived Immigrants Located in Chile? A Spatial Analysis of Census Data Using an Index of Multiple Deprivation from the Last Three Decades (1992-2012)

**DOI:** 10.1371/journal.pone.0146047

**Published:** 2016-01-12

**Authors:** Andrea Vasquez, Baltica Cabieses, Helena Tunstall

**Affiliations:** 1 Facultad de Medicina Clínica Alemana, Universidad del Desarrollo, Santiago, Chile; 2 National Research Center for Integrated Natural Disaster Management CIGIDEN, Fondap 15110017, School of Engineering, Pontificia Universidad Catolica de Chile, Santiago, Chile; 3 Department of Health Sciences, University of York, York, England, United Kingdom; 4 School of GeoSciences, University of Edinburgh, Edinburgh, Scotland, United Kingdom; University of Westminster, UNITED KINGDOM

## Abstract

**Introduction and Purpose of the Study:**

Immigrants in Chile have diverse characteristics and include socioeconomically deprived populations. The location of socioeconomically deprived immigrants is important for the development of public policy intelligence at the local and national levels but their areas of residence have not been mapped in Chile. This study explored the spatial distribution of socioeconomic deprivation among immigrants in Chile, 1992–2012, and compared it to the total population.

**Material and Methods:**

Areas with socioeconomically deprived populations were identified with a deprivation index which we developed modelled upon the Index of Multiple Deprivation (IMD) for England. Our IMD was based upon the indicators of unemployment, low educational level (primary) and disability from Census data at county level for the three decades 1992, 2002 and 2012, for 332, 339 and 343 counties respectively. We developed two versions of the IMD one based on disadvantage among the total population and another focused upon the circumstances of immigrants only. We generated a spatial representation of the IMD using GIS, for the overall IMD score and for each dimension of the index, separately. We also compared the immigrants´ IMD to the total population´s IMD using Pearson´s correlation test.

**Results:**

Results showed that socioeconomically deprived immigrants tended to be concentrated in counties in the northern and central area of Chile, in particular within the Metropolitan Region of Santiago. These were the same counties where there was the greatest concentration of socioeconomic deprivation for the total population during the same time periods. Since 1992 there have been significant change in the location of the socioeconomically deprived populations within the Metropolitan Region of Santiago with the highest IMD scores for both the total population and immigrants becoming increasingly concentrated in the central and eastern counties of the Region.

**Conclusion:**

This is the first study analysing the spatial distribution of socioeconomic deprivation among international immigrants and the total population in a Latin American country. Findings could inform policy makers about location of areas of higher need of social protection in Chile, for both immigrants and the total resident population in the country.

## Introduction

### Migration patterns in the world, in Latin America and in Chile

Immigration patterns have different waves and cycles over time. Worldwide, it is estimated that around 200 million people migrate every year [1, 2]. In Latin America and the Caribbean, some 25 million (about 4% of the total population) had migrated to a different country in 2011 [3, 4]. In general, the US is the preferred destination for migrants from Latin American and Caribbean nations [5, 6] and income differences between countries are one of the major reasons for these movement [5, 7]. There is also increasing migration within the Latin American region, predominantly the movement of people living in relatively less developed countries to more developed ones nearby [2].

**Fig 1:**
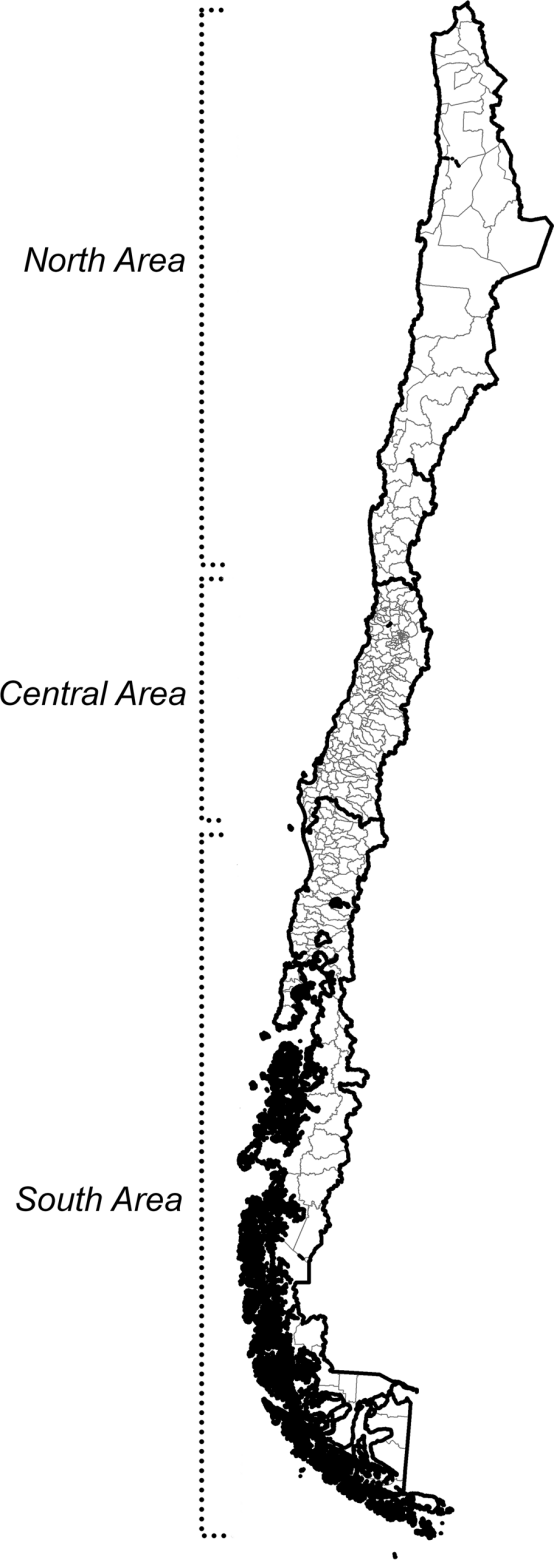
Map of Chile: its regions and counties. Source: Andrea Vasquez, Fondecyt 11130042, based on cartography provided by the National Institute of Statistics.

Chile is now defined as a high-income country with a Gross Domestic Product per capita of above $16,000 in 2014 (USD) [8]. In 2014 the country has a population of just over 16 million inhabitants [9], according to estimates from 2002 Census data, spread across continental, insular and Antarctic territory. The continental territory is comprised of north, central and south areas (Fig 1) divided administratively into 15 regions, which are also split into provinces and counties. Among the total population, 87% live in urban areas and just 13% in rural areas and forecasts suggest that population is continuing to concentrate in urban areas [10, 11]. Santiago, the city in Chile with the largest population, is located in the Central area. In 2012 the Metropolitan Region had a population of 6.7 million (based on Census 2002 data estimates), and has strongly service based employment. In the northern areas of Chile mining is central to the economy and the south area is focused upon manufacturing and extractive activities [12].

Chile in recent decades has experienced major economic and demographic changes, a progressive improvement of the health status of the population, a decline in infant and general mortality rates, and an increase in life expectancy [13, 14]. The health status of the Chilean population is now very similar to that of some more established high-income countries and better than many other Latin American nations [13, 15]. Chile´s economic growth and political stability in recent decades has made it increasingly attractive as a host country for immigrants, especially those from other Latin-American countries.

Patterns of immigration to Chile have changed over time. During the 1970s and 1980s immigration to Chile was mainly from Europe, Arabic countries and East Asia [16]. During the last two decades, however, rates of immigration from South American countries to Chile have increased [3]. The latest governmental figures indicate that currently Chile is experiencing a “new immigration” pattern with a majority of immigrants now of Latin American origin and of working age, seeking labor opportunities [2]. There has also been increasing female immigration in the Latin American region, including Chile [3, 17], in particular to work in manual and domestic services [18].

According to the latest national figures from the Department of Immigration, Chile currently has 441,000 immigrants, mainly from its three bordering countries, Peru, Argentina and Bolivia [19]. Immigrants represented about 2.5% of the total of 16 millions of people living in the country in 2014 [19].

Migration is a complex and dynamic process, influenced by a range of social processes and individual motivations, such as economic crisis, the search for better career and educational opportunities, and others. As a result of this complexity, there are several different theories proposed to explain the causes of international migration including the push and pull theory [20–22], the cumulative causation theory [20, 23], the global theory [24, 25], and behavioral theory [26]. *Cumulative causation* theory is one of the most frequently cited [27]. This dynamic theory is based on the “chain migration” concept which [20] suggests that before arriving in a country, immigrants often establish a social network in the foreign country, comprising relatives or friends from their country of origin. This social network may be concentrated in particular geographical places, and, consequently; such geographical areas (regions, provinces or neighborhoods) can become places of attraction for other potential migrants. One plausible effect of this theory is that socioeconomically deprived immigrants may concentrate in geographical areas [28, 29]. Such concentration might be associated with social cohesion and social support amongst immigrants [30, 31], but also with poverty, isolation, stigma and discrimination from local populations, and lack of connection with government authorities and other residents outside their area of residence [29, 32].

### Migration and socioeconomic deprivation: its spatial dimension

The possibility that immigrants in Chile are both socioeconomically deprived and spatially concentrated suggests the potential value to public policy of developing measures of deprivation describing their location within the country. In Chile two main approaches have been used to measure socioeconomically deprived populations in public policy. First, absolute poverty has been measured by the government using a poverty line equal to household income at or below $120 USD per capita and per month in urban areas. This poverty line is based on a basic guideline of food consumed per month. This method uses data from a socioeconomic survey completed in Chile each three years since 1985 and is represented at a regional spatial scale [33].

A second approach that has been used to measure deprivation in Chile has been developed as part of a Latin-American wide assessment of socioeconomically deprived populations. This approach is based upon Census indicators of a range of basic material and social needs, as indicated by sanitary systems, education, housing quality and overcrowding [34]. This index is developed for middle income Latin American countries in the region (Chile has been excluded since 2014) and is used to characterize and compare the different vulnerabilities in Latin American countries. Its’ main methodological limitations are that the variables vary between countries and over time (e.g. the definition of low level of education is different for each country and also varies over ten years). It is also available only at the national scale.

Outside of Chile in the last three decades indices describing area deprivation have been developed in several countries [35–37] to describe disadvantage within sub-national areas. Some of the first of these indices were developed in the UK using data from the 1981 Census to describe material deprivation in small areas. For example, the Townsend Deprivation index was developed to measure material disadvantage in neighborhood wards in England using four equally weighted census indicators: households without a car, overcrowded households, households not owner-occupied and persons unemployed [38]. Similarly, the Carstairs deprivation index was devised to measure material deprivation of neighborhood postcode sectors using four equally weighted measures from the 1981 Census in Scotland: households without a car, overcrowded households, low social class person and unemployment men [39].

More recently area deprivation indices have been developed that are intended to represent a broader range of types of deprivation. These indices, like the Latin-American indicators of unmet needs, assess not only material poverty, but also lack along a range of social dimensions [35–37] [40]. These newer indices have also been based upon data from a broader variety of sources including not only censuses, but also data from administrative sources and surveys. They have commonly been used to describe neighbourhoods but have also been devised for large areas [41]. Deprivation indices have also been developed to identify deprivation not only among the general population [35] but also for specific groups such children [42] and elderly people [43], or specific types of deprivation such as health deprivation [36].

In this study, we have drawn on principles underlying the English Index of Multiple Deprivation (IMD) as a model for our index [41]. IMD has been used extensively in England for spatial analysis related to material deprivation, health and social policy [41]. The English Index of Multiple Deprivation (2010) conceptualizes area deprivation as multi-dimensional and is a multi-dimensional index of deprivation. It includes the following dimensions: 1) Income (two measures: unemployment of the head of the household and household living below the poverty line), 2) Employment (unique measure of three categories: unemployment, sickness or disability), 3) Health and disability (four measures: years of potential life lost, comparative illness and disability ratio, acute morbidity, mood and anxiety disorders), 4) Education, skills and training (measured by last completed education level), 5) Barriers to housing and services (physical and financial access to housing), 6) Crime, any of the following measures: violence, burglary, theft, criminal damage, 7) Living environment (measures the quality of individuals’ immediate surroundings both within and outside the home) [41].

### The purpose of this study

While some previous studies have explored the living conditions and health needs of groups of international immigrants in specific counties in Chile like Santiago and Arica [44–46], their experience of deprivation has not been assessed across the whole of the country. We aimed to fill this research gap in Chile by describing the spatial location of deprived immigrants in all regions. In particular, further spatial information is required to develop a strategic plan to support socioeconomically deprived immigrants in Chile. Our research is intended to support this policy goal.

The main objective of this study was to identify areas with high concentrations of socioeconomically deprived immigrants. We also aimed to compare the location of socioeconomically deprived immigrants to that of socioeconomically deprived people in the wider population. Lastly, our objective was to assess how the geography of this socioeconomically deprived population has changed over time. We aimed to do this by creating indices to measure socioeconomic deprivation, drawing on previous geographical methodologies developed to measure area disadvantage from the English IMD. Through these analyses we hoped to make a unique contribution to knowledge regarding international migration in Chile, which could also serve as a model for other countries in Latin America.

## Materials and Methods

We developed our IMD for two different populations, immigrants and total population, at the county level, over three decades. This work was conducted using freely available Census data from Chile, which can be downloaded from this Web Page (1992, 2002): http://www.ine.cl/canales/chile_estadistico/censos/censo_poblacion_vivienda.phpCensus data for 2012 is supported in a SQL database, physically available in Chilean Institute of Statistics. For getting more information about the data availability for censuses 1992, 2002 and 2012 see the letters provided by the National Institute of Statistics in Chile: S1 File, S2 File, S3 File and S4 File. For Censuses 1992, 2002 and 2012, databases and cartography are available only upon request. This is public data about census databases, but is not free downloadable and you have to seek the information through the corresponding public institution, according to the 18th Article in the Chilean Law No. 20.285 about access to public information. For accessing to the data of cartography and censuses databases used and cited in the manuscript, from 1992 to 2012, researchers must send an e-mail to the National Institute of Statistics (Chile): transparencia@ine.cl in Spanish language.

This study is part of the Fondecyt grant number 11130042 (2013–2017). The protocol was reviewed by (i) the Ethics Committee at the Faculty of Medicine Clínica Alemana Universidad del Desarrollo de Chile and (ii) The Ethics Committee at Conicyt Chile (National Commission for Scientific and Technological Research, Chilean government).

### Data Source

#### The Census in Chile

We calculated our Index of Multiple Deprivation (IMD) based on available variables from the Chilean Censuses in 1992, 2002 and 2012. The Census in Chile have taken place with the recommended periodicity (every 10 years) since 1835 [47].

Censuses seek to characterize the total population and housing and are often used for public policy development. The 1992 and 2002 Censuses in Chile were largely representative of the total population, but the latest Census of 2012 had some methodological problems and showed a higher proportion of missing data than the previous ones (9.6% missing values in 2012 versus 3.8% in 2002 and 1.9% in 1992) (see Table 1). This problem has reduced the value of the 2012 Census for research and the development of public policies [48], but the Census still represents the most comprehensive national source of data for the country for that year. We performed our spatial analysis with this limitation in mind, accepting the higher chance of bias of 2012 Census compared to 1992 and 2002. Findings from this study for 2012 must be interpreted, therefore, as exploratory rather than definitive.

**Table 1:** Sample of the Chilean Censuses 1992, 2002, 2012. Source: Andrea Vasquez, Fondecyt 11130042.

Census per year	1992	2002	2012
**Considered counties**	332	339	343
**Counted people (Chile)**	13.348.401	15.116.436	16.341.929
**International immigrants (Chile)**	114.498	197.929	324.074
**Missed data % (Chile)**	1,9	3,8	9,6
**Missed data % (Latin America average)**	5,3	4,1	-

#### Selection of spatial scale

We collected the data for each variable from the Censuses at the county level for the entire national territory using software Redatam version 5.0 as provided by the Chilean Institute of National Statistics.

Data was available for counties, census tracts and census blocks. Although many deprivation indices have adopted smaller spatial areas than county [49, 50], we chose the this scale because we wanted initially to focus upon the identification of broad patterns of deprivation to support the development of public policies at the national and regional level. Our index could subsequently be produced at a smaller scale–like IMD in England which is calculated for neighborhoods and larger local government areas.

#### Definition of immigrant status

We defined immigrants as all people who self-reported in the Census that they were born in another country. In the Census questionnaire in each of the three censuses immigrants are described through two main questions. The first one is focused on permanent immigrants and asks in which country they were born. The Census is anonymous and unlike immigration data from the Government´s Immigration and Foreigners Service, which records only legal immigration, should include undocumented immigrants. There is less than 2% missing data for this question regarding country of birth in each of the three censuses.

The second question related to migration concerns where residents lived five years ago. This question could provide information regarding the movements of immigrants, and also the movements of the wider Chilean population. However, this question has some of the highest levels of missing data in the Census questionnaire resulting from non-response (about 40% missing data). For this reason we chose to use only the question focused on permanent immigrants living in Chile.

### Measuring socioeconomic deprivation: the IMD

#### Measuring IMD for immigrants

In this study we estimated the IMD based on three key dimensions of deprivation that can be measured with data from the Chilean Census: low level of education, disability and unemployment [49–52]. Other important aspects of deprivation, such as quality of life, crime, and access to services or income, which are part of the English Index of Multiple Deprivation are not regularly included in the Census questionnaire in Chile. In addition, other potential sources of data for these dimensions of deprivation were not available in Chile for all the years of the study. Table 2 shows the key Census questions on which we based our indices.

**Table 2:** Variables for estimating the IMD and its correspondence with the Chilean Census questionnaire. Source: Andrea Vasquez, Fondecyt 11130042.

Variable	Census’s question	Recodification strategy for analysis
Low level of education	Last course attended in formal education (Categories: not assistance to school until elementary education, 8^th^ grade)	Sum of total population for each county included in the following categories: from people who did not study until people who ended their formal education when they ended the elementary education (k-8^th^).
Employment	Employment situation (Categories: wage earners, independent, housework without income, unemployed (person who worked before but now is looking for a job)).	Total quantity of unemployment per county.
Disability	Condition of disability considering all population. Categories: dumbness, deafness, blindness, motor disability, mental disability	Sum of total quantity of all categories included in condition of disability per county.

Each variable in the IMD, employment, health and education, was given an equal weight, as shown in the Eq 1.

$$ImmigrantsIMD=\left[\left(Unemployed_{immigrants}*0,\bar{3}\right)+\left(Disabled_{immigrants}*0,\bar{3}\right)+\left(LowLevelEducation_{immigrants}*0,\bar{3}\right)\right]$$ Eq 1

The dimensions of deprivation included in the original IMD from England [41] have unique weights. For this first estimation of IMD in Chile, we felt however that a more cautious approach to weighting was appropriate. We therefore followed the weighting methodology of the UK Census based Townsend [38] and Carstairs [39] indices and used equal weights for all deprivation indicators. We completed sensitivity testing to explore to what extent the Chilean IMD scores would vary across counties if different weights were used for each dimension of deprivation. This testing found broadly similar counties emerged with the highest scores for deprivation when different weighting strategies were used. This reflects the high degree of correlation between the three deprivation measures (these relationships area described further in the results). Future research could assess weighting further to determine the most appropriate approach to deprivation weighting for areas in Chile.

Each variable was normalized based on a linear distribution of the variable (*Zscore*) (see Eq 2).

$$NormalizedValue(NV)=\left[\left(X_{i}-X_{\min}\right)*\left(\frac{1}{X_{\max}-X_{\min}}\right)\right]$$ Eq 2

Then, we added all three factors, which generated a normalized IMD variable with possible values between 0 and 1. To simplify interpretation, we multiplied this value by 100, in order to convert it to percentages. The IMD estimate then ran from 0 to 100.

#### Spatial units over time

In order to make the Census data comparable over time we required a consistent geography in each time period. However there were small changes in the county geography between Censuses. The IMD was therefore calculated for 332 counties for 1992, 339 in 2002 and 343 in 2012. The change in numbers occurred because some counties were subdivided over time following population growth. Between 1992 and 2002 seven counties were subdivided (2% over the total counties) and between 2002 and 2012 three were subdivided (0.8% of the total counties). Therefore changes to county boundaries affected less than 3% of the total territory under study.

It is also relevant to note that the total counties considered in this study include the continental territory only while Antarctica and insular counties were excluded from this study. This was because cartographic data provided by the Chilean Statistics Institute is only available for the continental territory. Hence, we estimated the IMD for the two study populations for the entire continental territory and for each county in the three time points: 1992, 2002, and 2012.

### Data analysis

We first mapped the total international immigrant population in Chile at the county level for the three periods under study (1992, 2002, and 2012) using the ArcGis 10.0 software. Then we mapped the IMD indices for both immigrants and the total population. The IMD component variables were then assessed in separate maps. We ranked counties by immigrant IMD score and identified areas with the greatest concentration of socioeconomically deprived immigrants. Correlation testing was then carried out, as described in Table 3, aimed at testing associations between IMD for immigrants and the total population, between socioeconomically deprived immigrants and total immigrants, between specific dimensions of the IMD and between years of Census data. Correlations were tested at a 95% confidence level using the Pearson correlation test.

**Table 3:** Variables included for correlation. Source: Andrea Vasquez, Fondecyt 11130042.

Variables tested for correlation
Immigrant IMD and immigrant population
IMD of total population and immigrant population
IMD of total population and immigrant IMD
Immigrant IMD and unemployment in total population
Immigrant IMD and low level of education in total population
Immigrants IMD and disability in total population
Immigrant disability and unemployed immigrants
Disabled immigrants and low level of education in immigrants
Unemployed immigrants and low level of education in immigrants

## Results

During the last three decades the international immigrant population has grown in Chile, from 0.9% of the total population in 1992, to 1.3% in 2002 and reaching 2.0% of the total population in 2012.

The IMD for international immigrants in Chile showed a range between 0.01 and 86.89 across different counties (mean 3.4, s.d. 10.1 for the three years in study). The same Figure for the total population was between 0.02 and 90.0 (mean 11.5, s.d. 15.3). When comparing overall IMD averages per year for each population, the mean IMD in 1992 for immigrants was 3.9 versus a mean of 11.5 in the total population in the same time period. This difference was maintained in 2002 with 3.6 for immigrants versus 10.1 for total population, but the IMD decreased in 2012 to 2.7 for immigrants and 8.7 for the total population. So, notably, across the three decades of study the total population has a higher average socioeconomic deprivation than immigrants.

### The spatial distribution of total immigrants

**Fig 2:**
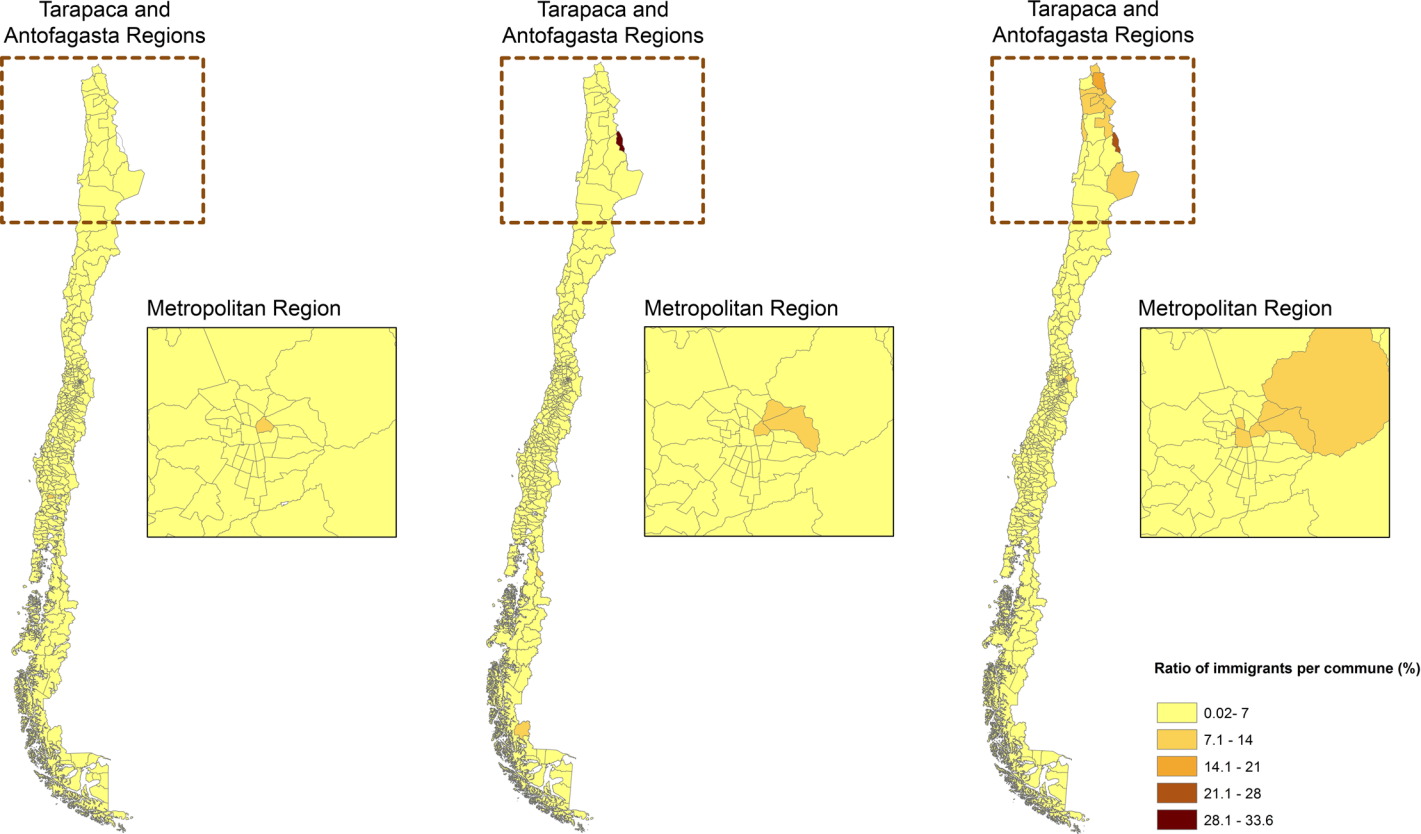
The spatial distribution of the total international immigrant population in Chile by counties in 1992, 2002 and 2012 (in that order from left to right in the Figure). The Metropolitan region equals to Santiago city. Source: Andrea Vasquez, Fondecyt 11130042, based on cartography provided by the National Institute of Statistics.

In 1992 international immigrants were concentrated in parts of the central area of Chile, in particular the Metropolitan Region of Santiago. This pattern had changed in 2002. By this year international immigrants not only appeared to have expanded into new counties within the central area of Chile, but there were also significant populations in the north of Chile, particularly in counties with high production of copper and commercial services (Fig 2). In 2012 international immigrants continued their territorial expansion, and were found to be more dispersed than in the previous two decades. Despite this modest degree of dispersion compared to previous Censuses, they still remained most concentrated in the north and central areas of Chile as a whole.

### The distribution of the Index of Multiple Deprivation for immigrants and total population

**Fig 3:**
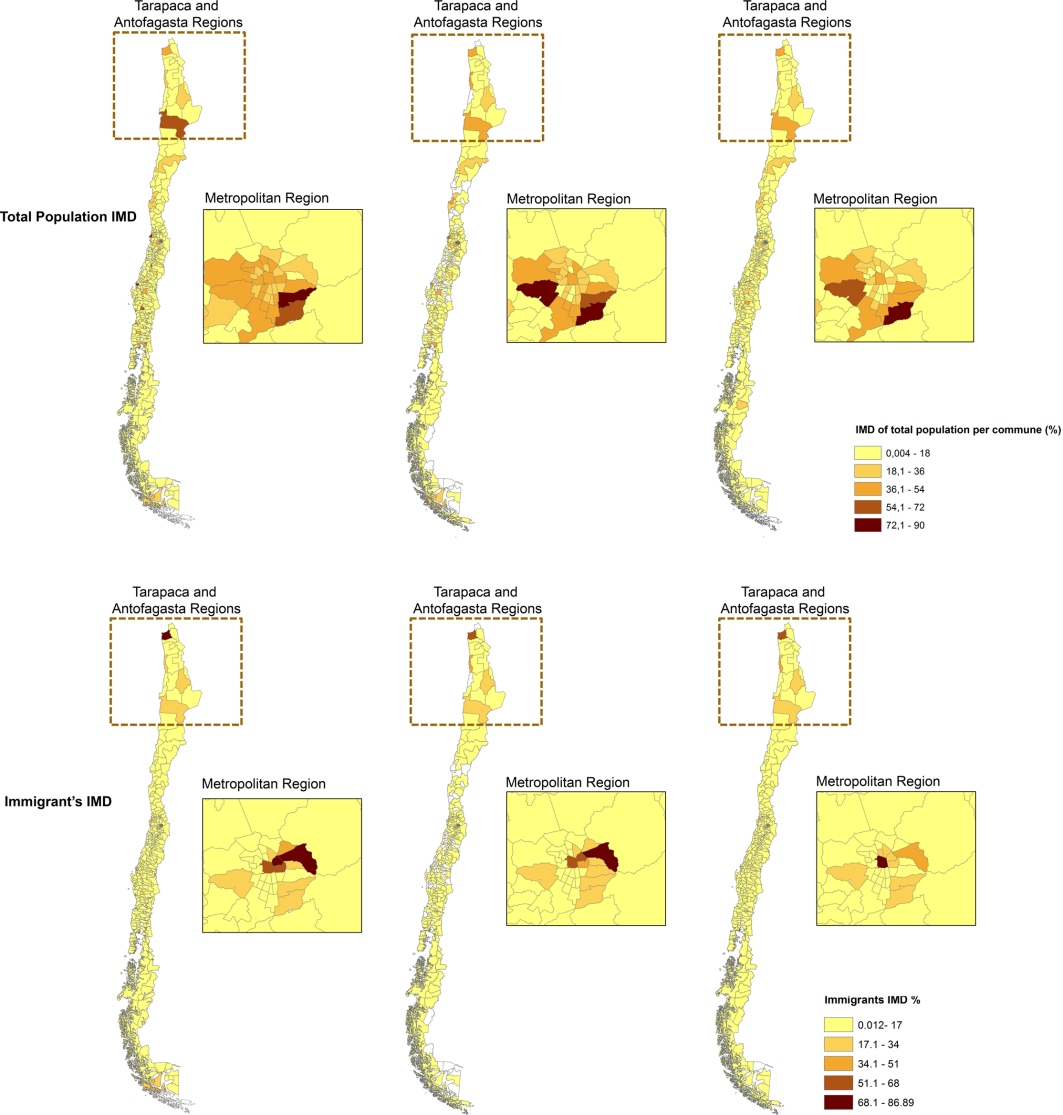
The spatial distribution of the IMD for the total population (above) and the immigrant population (below) by counties in 1992, 2002 and 2012 (in that order from left to right in the Figure). The Metropolitan region equals to Santiago city. Source: Andrea Vasquez, Fondecyt 11130042, based on cartography provided by the National Institute of Statistics.

The IMD scores for the country in 1992, 2002 and 2012 were mapped for each county and appear in Fig 3. Despite the territorial dispersal of international immigrants as a whole observed in the last three decades, we found socioeconomically deprived immigrants remained concentrated during the time period in north and central areas, especially within the Metropolitan Region of Santiago. Among the total population there were also high concentrations of deprived populations in north and central areas. The spatial distribution of the IMD for the total population was however more dispersed than that of the IMD for international immigrants. Within the Metropolitan Region of Santiago the location of socioeconomically deprived people among the total population and immigrants has changed significantly over time as they have become more concentrated in central and eastern areas of the Metropolitan Region after 1992.

**Fig 4:**
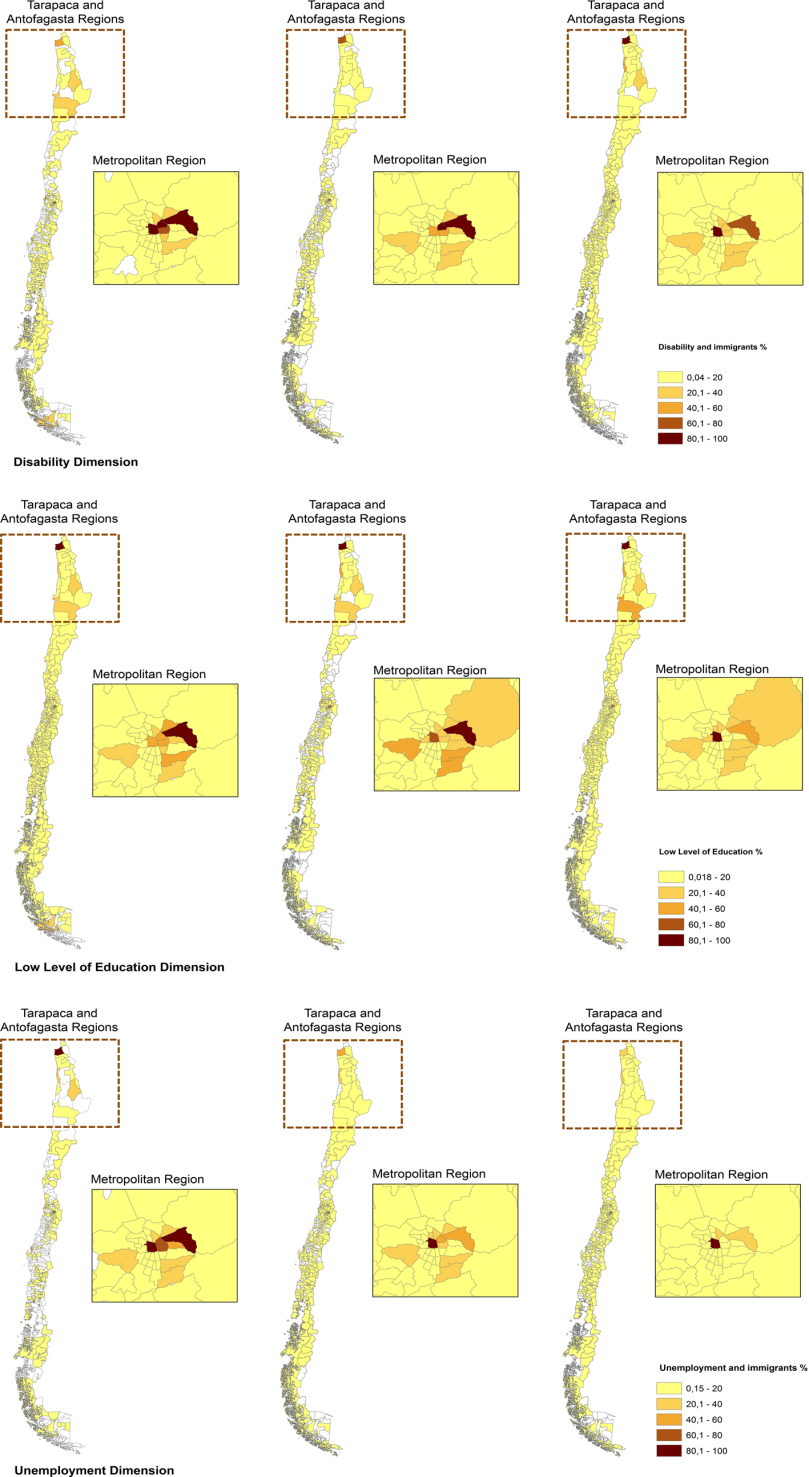
The spatial distribution of the IMD dimensions separately for the immigrant population, by counties in 1992, 2002 and 2012 (in that order from left to right in the Figure). Source: Andrea Vasquez, Fondecyt 11130042, based on cartography provided by the National Institute of Statistics.

We also explored the spatial distribution of the dimensions of the IMD among the international immigrant population. Overall, we found a fairly similar pattern for each dimension of the total IMD score for both immigrants and the total population. The three tended to be concentrated in the same areas of the north and central regions of the country. Nonetheless, it should be noted that each dimension of the IMD showed a greater spatial dispersion on its own, so the combination of the three together indicated a unique group of highly socioeconomically deprived counties in the country. These counties would have not been identified if the three dimensions were assessed separately (see Fig 4).

### Ranking the counties with the highest scores of immigrant IMD

We ranked the top 10 counties with the highest socioeconomic deprivation among immigrants in each decade. Table 4 displays this ranking, which highlights the consistency over time of three counties with high levels of socioeconomic deprivation: Las Condes, Arica and Santiago. The Table also demonstrates how the distribution of immigrants has evolved in the past three decades. For example, we observed a growing concentration of both total and socioeconomically deprived immigrants in some specific counties of the Metropolitan Region over time, such as Maipú, Independencia, and Recoleta, and other specific counties in the north area of Chile, such as: Calama and Antofagasta. We can also observe the decline in socioeconomic deprivation in counties such as Viña del Mar and Providencia over time. These areas are now known as relatively wealthy counties. Socioeconomically deprived immigrants, therefore, seemed to have moved away from these wealthy counties over time, but have remained fairly stable in less affluent counties like Arica during the same period of time.

**Table 4:** Ranking the counties with the highest scores of immigrant IMD during the three last decades. Source: Andrea Vasquez, Fondecyt 11130042.

Ranking	County 1992	IMD 1992	County 2002	IMD 2002	County 2012	IMD 2012
**1**	Las Condes	86,2	Las Condes	73,1	Santiago	86,9
**2**	Arica	75,3	Santiago	66,2	Arica	61,7
**3**	Providencia	68,0	Arica	61,1	Las Condes	44,9
**4**	Santiago	66,2	Providencia	51,4	Iquique	37,3
**5**	Ñuñoa	57,4	Ñuñoa	36,5	Recoleta	26,3
**6**	Viña del Mar	50,0	Iquique	34,6	Antofagasta	24,5
**7**	Vitacura	34,2	Viña del Mar	33,0	Calama	23,0
**8**	La Reina	29,2	La Florida	32,7	Providencia	21,3
**9**	La Florida	29,2	Maipú	31,3	Viña del Mar	19,6
**10**	Valparaíso	28,8	Puente Alto	29,9	Puente Alto	19,4

### Correlations between the IMD and immigrant variables

Table 5 displays the correlations between the IMD and immigrant variables. We found a significant correlation between all variables. The highest correlation was between immigrants IMD and the total immigrant population which was 0.96 in all decades. The lowest correlation was found between IMD for total population and immigrant population; 0.48–0.55 over the three decades. This indicated that the immigrant population were often, but not always, concentrated in areas with high levels of deprivation among the total population. The correlation between IMD for immigrants and IMD for total population was between 0.54 and 0.66, with the lowest correlation in 2012. Immigrant IMD was also fairly highly correlated with each of the components of the total population IMD. This suggests both significant overlap and some differences in the location of socioeconomically deprived people in the immigrant and total population. Finally, there were very high levels of correlation between all three dimensions of immigrant socioeconomic deprivation in all three decades.

**Table 5:** Correlation estimates of IMDs between populations and between dimensions. Source: Andrea Vasquez, Fondecyt 11130042.

Correlation tests	1992	P-value (n = 331)	2002	P-value (n = 339)	2012	P-value (n = 343)
Immigrant IMD / Immigrant population	0.96	< 0.00001	0.96	< 0.00001	0.96	< 0.00001
Total population IMD / Immigrant population	0.52	< 0.00001	0.55	< 0.00001	0.48	< 0.00001
Total population IMD/ Immigrant IMD	0.62	< 0.00001	0.66	< 0.00001	0.54	< 0.00001
Immigrant IMD / Total population unemployment	0.63	< 0.00001	0.68	< 0.00001	0.57	< 0.00001
Immigrant IMD / Total population education	0.57	< 0.00001	0.59	< 0.00001	0.49	< 0.00001
Immigrant IMD/ Total population disability	0.63	< 0.00001	0.67	< 0.00001	0.53	< 0.00001
Immigrant disability / Immigrant unemployment	0.93	< 0.00001	0.87	< 0.00001	0.86	< 0.00001
Immigrant disability / Immigrant education	0.9	< 0.00001	0.86	< 0.00001	0.95	< 0.00001
Immigrant unemployment / Immigrant education	0.96	< 0.00001	0.88	< 0.00001	0.88	< 0.00001

## Discussion

### Summary of main findings

In this study we found that the number of immigrants and their spatial distribution across the continental territory of the country has evolved in the past three decades. In 1992 immigrants were predominantly located in central regions of the country, especially in the Metropolitan Region of Santiago. However, during 2002 and 2012 as the numbers of immigrants increased Chile experienced two developments: (i) a higher concentration of immigrants in some specific counties in the center of the country like Santiago, possibly due to more job opportunities; and (ii) a higher concentration of immigrants in the northern area of Chile, especially in the Tarapacá and Antofagasta regions, possibly due to geographical proximity to sources of immigrants from bordering countries like Peru and Bolivia.

Our IMD indicated that immigrants and socioeconomically deprived immigrants tended to be more concentrated in counties in the northern and central area of Chile, especially within the Metropolitan Area of Santiago, the main Region of Chile. These were often the same counties where people in the total population with socioeconomic deprivation were most concentrated during the same time periods. We observed a growing concentration of both total and socioeconomically deprived immigrants in some specific counties of the Metropolitan Region over time, such as Maipú, Independencia, and Recoleta, and other specific counties in the north area of Chile.

### Contrasting findings with theory and previous research

According to our findings, there is an association between higher concentration of socioeconomic deprivation for the total population and international immigrants within counties in many areas of the country. Based on the social chain theory of migration, we could argue that these counties have some social characteristics that draw immigrants as well as job or educational opportunities. They might be social networks that immigrants have developed through the migration process with relatives or friends, which are particularly important when making the decision on whether to migrate to Chile. In addition, irrespective of the social networks immigrants in the country, once they have arrived they may tend to move to specific counties that have become denser with immigrant population over time.

We found that counties with high socioeconomic deprivation for the total population often had high socioeconomic deprivation for immigrants (i.e. high and significant correlation estimates between immigrants IMD and each dimension of IMD for the total population). This implies that the concentration of socioeconomic deprivation is spatially distributed in Chile, not only for international immigrants but also for the total socioeconomically deprived population in the country. This needs further consideration and research to develop more robust, effective public policy strategies targeting these groups with significant socioeconomic deprivation.

As shown in other studies of deprivation [49–52], deprivation indices are often used to study the more deprived areas within a large city. In this study we wanted to describe immigrant’s IMD across the entire continental territory of the country. This description in Chile should inform public policy practitioners and health practitioners that the socioeconomic deprivation among immigrants affects many counties, but the more socioeconomically deprived immigrants are concentrated in some specific areas. Studies in the past have explored the living conditions and health needs of groups of international immigrants in specific counties in the counties like Santiago and Arica [44–46]. This study provides unique and novel findings on the spatial distribution of socioeconomic deprivation of international migrants and the total population across the country as a whole. It identifies the existence of some specific counties with high concentrations of socioeconomic deprivation among immigrants and the total population in Chile. This highlights the need for both local and national strategies to prevent and reduce socioeconomic deprivation among both immigrants and the total population.

### Study’s strengths

This is the first study exploring the IMD for each county in the continental territory of Chile. Census data used in this study is the most representative source describing the total population. While the 2012 dataset has a greater level of bias than 1992 and 2002 (with 9.6% missed data at the national level) it still compares well with some previous censuses in Chile (the greatest missing data was in 1952 and 1970 Census of Housing and Population) and with the error estimates from Censuses conducted in other Latin American countries during the 1990s [48].

We created a deprivation index drawing on the conceptualization of the English IMD and despite its limitations, this scale is a novel contribution to research in Chile and Latin America. It adds new knowledge for improving policy and practice, especially by supporting a spatial dimension to the monitoring of socioeconomic deprivation in the country in the future.

In this study, we conceptualized and measured socioeconomic deprivation as a complex, multidimensional social construct, and we measured it not only in a single point in time but across three decades. This has allowed us to assess general changes in patterns of socioeconomic deprivation over a 30-year period of time in the country.

### Study’s weaknesses

There are some limitations to address in this study. First, the counties for which data was available varied in each Census because of the administrative changes that created 7 new counties in the period 1992–2002 and 3 new counties for 2002–2012 decade. This results in methodological weakness when comparing socioeconomic deprivation between counties over time. This issue makes comparison over time difficult for some specific counties that were measured one year and not the other. This however effected only 10 counties overall, which are a fairly small proportion of the total dataset (3%). The case of ‘Alto Hospicio’ county is the most important for the current study, because of its location in the north area with one of the highest immigrant populations in Chile, as seen in results.

Another weakness stems from the limitations of migration data in the Chilean Census. We focused on data regarding country of birth only because this census question had low levels of missing data. A further immigration question in the Census asked where residents lived five years ago and could have been used to assess recent immigrants who may be particularly pertinent to understanding the changing nature of the immigrant population immigrants in Chile. However, we did not assess this data because of high rates of non-response.

While the English IMD describes seven dimensions of deprivation we considered just three dimensions. This was due to lack of availability of other measures from relevant population-based sources at the time this study was conducted, particularly for the year of interest (1992, 2002 and 2012). Finally, as the value of Census 2012 is limited by missing data, we still require the confirmation of results from a new more fully representative Census in Chile.

### Implications for policy

The IMDs we have developed for immigrants and total population highlight the location of socioeconomically deprived populations in the country. The spatial analysis of socioeconomic deprivation provides an opportunity for focusing social public policy upon more deprived counties within Chile. The availability of knowledge regarding socioeconomic deprivation at county level can support further local disaggregation, which would allow more detailed territorial management and public policy application in Chile. In this way, it could be possible to use this county level information as a base layer for local management to focus effort and resources to support socioeconomically deprived local populations. This IMD could provide crucial information to tackle health and social issues, allowing the prioritization of counties that are more socioeconomically deprived than others.

### Future research

A future version of this index in Chile could consider further dimensions of multiple deprivation and could also be applied at a more detailed spatial scale. We have considered variables corresponding to demographic and socioeconomic deprivation, but new dimensions could be added. These could potentially include distance and access to services, focused in particular on access to healthcare facilities and crime. The index could also incorporate data linked to the poverty line.

In addition, future research could measure socioeconomic deprivation not only at the county level, but study in depth what is happening at a more detailed level, like the neighborhood, used in the original IMD of England. This IMD at a more detailed spatial scale, could be based upon the census block used in the Chilean Census. Although, data describing some aspects of socioeconomic deprivation (e.g. crime) are not available at this scale.

We also need to complete further work assessing the best approaches to weighting different aspects of socioeconomic deprivation in an IMD. This analysis should determine which variables are more important in defining socioeconomic deprivation and how to develop a different weight for each variable.

Future work could support the development of the multiple deprivation index at different scales with data that can be updated periodically from public information and reports, allowing the utilization of this index by national and local government. Socioeconomic deprivation is a complex and dynamic concept and so this index will need to be updated to support future policy.

## Conclusions

Our main research questions concerned, firstly, where socioeconomically deprived immigrants have concentrated spatially over the last three decades, and secondly if their location was correlated with the location of socioeconomically deprived people among the total population. Through this analysis we aimed at providing unique knowledge in the field of international migration in the Southern cone of Latin America. We created a variation of the English IMD, using the indicators of unemployment, low educational level and disability that were available from Census data in three decades, 1992, 2002 and 2012.

We found that socioeconomically deprived immigrants tended to be more concentrated in counties in the northern and central area of Chile. These were often the same counties where there was concentrated socioeconomic deprivation for the total population during the same time periods. This is the first study analysing the spatial distribution of socioeconomic deprivation of international immigrants and the total population in a Latin American country. Findings could inform policy makers on the location of areas with populations at higher need of social protection in Chile and support national and local polices to address these needs among both among immigrants and the total resident population in the country. These spatially targeted policies may be especially important for immigrants, due to their population growth in the last three decades and their strong spatial concentration in some regions of Chile.

## Supporting Information

S1 File This is the S1 Letter for Censuses 1992 and 2002 Spanish. This is the original letter provided by the National Institute of Statistics in Chile about how to get the information from Census 1992 and 2002.
(PDF)

S2 File This is the S2 Letter for Censuses 1992 and 2002 English. This is the translated letter into English provided by the National Institute of Statistics in Chile about how to get the information from Census 1992 and 2002.
(PDF)

S3 File This is the S3 Letter for Census 2012 Spanish. This is the original letter provided by the National Institute of Statistics in Chile about how to get the information from Census 2012.
(PDF)

S4 File This is the S4 Letter for Census 2012 English. This is the translated letter into English provided by the National Institute of Statistics in Chile about how to get the information from Census 2012.
(PDF)
