# Supplementary material for: Where Are Socioeconomically Deprived Immigrants Located in Chile? A Spatial Analysis of Census Data Using an Index of Multiple Deprivation from the Last Three Decades (1992-2012)
Source: PLoS One. 2016 Jan 12;11(1):e0146047. doi: 10.1371/journal.pone.0146047 (PMC4710505; doi:10.1371/journal.pone.0146047)
Supplement: S3 File — This is the original letter provided by the National Institute of Statistics in Chile about how to get the information from Census 2012. (PDF) [file pone.0146047.s003.pdf]

ORD. N°0896.

ANT.: Solicitud AH007P-0002786 de fecha 20.10.2014.

MAT.: Informa sobre costos de reproducción.

Santiago, 22 De Octubre de 2014

Señora
Andrea Vásquez González.
Membrillar 45.
La Florida.

Estimado señora:

Junto con saludarle cordialmente, le comunicamos que la información requerida por UD., en la solicitud N° AH007P-0002786 se encuentra disponible para la entrega en el formato y el medio por usted señalado.

No obstante, y de acuerdo al artículo 18 de la Ley N°20.285 Sobre Acceso a la Información Pública, le notificamos que para hacer efectiva la entrega es necesario que Ud. realice el pago de $ 450, por Cartografía Precenso 2011 formato (.GDB) y $ 530 por DVD Base de Datos Censo 2012, formato SQL, valor correspondiente a los costos de reproducción de la información, que han sido fijados por nuestra Institución, de acuerdo a la Resolución Exenta N° 3.050, de fecha 07 de Octubre de 2013.

El pago deberá realizarse en las dependencias del Instituto Nacional de Estadísticas, ubicadas en Paseo Bulnes N° 418, Piso 1, en el Subdepto. de Información Ciudadana, horario de atención: lunes a viernes de 09:00 a 14:00 hrs. en Santiago.

En consideración a lo anterior, le informamos que usted tendrá 30 días hábiles para efectuar el pago del importe señalado. No obstante, le solicitamos confirmar la aceptación de su requerimiento al correo transparencia@ine.cl, para que desde ese momento, nuestros profesionales puedan realizar la reproducción de la información por el medio señalado por usted.

En el caso que no pueda cubrir los costos de reproducción establecidos en la normativa legal vigente, podrá acceder a la información, proporcionando el soporte u otro dispositivo que permita su almacenamiento, en cuyo caso la reproducción será totalmente gratuita, siendo necesario concordar con el Subdepto. de Información Ciudadana, el día y horario para su realización.

Vencido el plazo legal que este Servicio tiene para la entrega de la información, o denegada ésta, el requirente tiene derecho a recurrir ante el Consejo para la Transparencia, solicitando amparo a su derecho de acceso a la información conforme lo establece el artículo 24 de la Ley 20.285 Sobre Acceso a la Información Pública.

Le saluda atentamente,

INSTITUTO NACIONAL DE ESTADÍSTICAS
