# Supplementary material for: Where Are Socioeconomically Deprived Immigrants Located in Chile? A Spatial Analysis of Census Data Using an Index of Multiple Deprivation from the Last Three Decades (1992-2012)
Source: PLoS One. 2016 Jan 12;11(1):e0146047. doi: 10.1371/journal.pone.0146047 (PMC4710505; doi:10.1371/journal.pone.0146047)
Supplement: S4 File — This is the translated letter into English provided by the National Institute of Statistics in Chile about how to get the information from Census 2012. (PDF) [file pone.0146047.s004.pdf]

ORD. N°0896

ANT.: Requirement AH007P-0002786 dated 20.10.2014.

MAT.: Notification about reproduction costs

Santiago, October 22nd 2014

Mrs
Andrea Vásquez González.
Membrillar 45.
La Florida.

Dear Mrs Vásquez:

We are pleasant to express our regards to you and to tell you that the information asked by you in the request Number AH007P-0002786 is available in the format and support defined by you.

Nevertheless and in order to the Law Number 20.285, Article number 18th about access to public information, we inform that for giving the information to you is necessary a payment of $450 (USD 0,75) and $530 (USD 0,8). These payments will include the reproduction cost of the following information: Pre Census 2011 cartography in format .GDB (Geodatabase) and a DVD that includes the Census 2012 database in SQL format. This reproduction cost regarding public information has been defined by our institution according the Exempt Resolution Number 3.050, dated October 7th, 2013.

The aforementioned payment should be done in the National Institute of Statistics, located at Paseo Bulnes N° 418, Piso 1, Sub-Department of Civic Information, schedule for citizen attention: Monday-Friday from 09:00 to 14:00 in Santiago.

Considering the previous information, we inform that you will have 30 weekdays for paying the reproduction cost. Despite we encourage you to confirm the acceptance of this requirement to the following e-mail address: transparencia@ine.cl, in order to collect all the information asked by you in the supporting media (DVD) by our staff.

In case you cannot cover the reproduction costs established by the legal basis applied to public information, you can access to this information providing your own device to storage your required information. In that case, the reproduction costs will be free of charge. The only requirement for getting the information through this way is to make an appointment with the Sub-department of city information.

Once expired the legal period applicable to this Service for giving you the required information, or in case of denied access to the information, you have the right to ask to the Council of Transparency about protection to your right of accessing to public information according what Law 20.285, article Number 24 say about the Access to the Public Information.

Sincerely yours,

National Institute of Statistics
